# Productivity changes during the COVID-19 pandemic and its associated risk factors

**DOI:** 10.5271/sjweh.4237

**Published:** 2025-09-01

**Authors:** Guilherme Monteiro Sanchez Dalla Riva, Sander K R van Zon, Patricia Ots, Gerard van den Berg, Lifelines Corona Research Initiative, Sandra Brouwer, Raun van Ooijen

**Affiliations:** 1 University of Groningen, University Medical Center Groningen, Department of Health Sciences, Community and Occupational Medicine, Groningen, The Netherlands.; 2 Netherlands Organisation for Applied Scientific Research, Unit Health & Work, Leiden, The Netherlands.; 3 University of Groningen, Department Economics and Business, Groningen, The Netherlands.; 4 Department of Epidemiology, University of Groningen, University Medical Center Groningen, Groningen, The Netherlands

**Keywords:** unemployment, sickness absence, working hours, quality of work, cohort study

## Abstract

**Objective:**

This study aimed to investigate productivity loss during the COVID-19 pandemic and identify risk factors by examining indicators of work productivity loss in a population-based cohort in The Netherlands.

**Methods:**

Longitudinal data from the Lifelines COVID-19 cohort were used, enriched with registry data from Statistics Netherlands. Data of N=11 462 workers were collected from 2020–2022. Productivity loss was measured using four indicators: unemployment, sickness absence rate, loss of work hours, and loss of work quality. Generalized estimating equations were used to examine the association between socioeconomic, health-, and work-related characteristics and the four indicators.

**Results:**

Unemployment remained low (<0.5%) throughout the pandemic. In contrast, prevalence of sickness absence, reduction of work hours and work quality peaked at 8.7%, 15%, and 4.7%, respectively. Critical work was associated with higher odds of sickness absence and quality loss, but lower odds of unemployment and loss of hours. Younger age and recent COVID-19 were associated with higher odds of sickness absence, loss of work hours and quality. Chronic health conditions were associated with higher odds of sickness absence and quality loss. Having children was associated with lower odds of unemployment and loss of hours.

**Conclusion:**

Despite low unemployment rates, productivity loss was observed at other indicators: sickness absence, loss of hours and quality. In addition, productivity was lost unequally among groups. When preparing for future crises, attention should be paid to broader indicators of productivity loss among different groups. Findings may help for offering targeted interventions to minimize losses in productivity and protect higher risk groups of workers

The COVID-19 pandemic led to a global economic and health crisis with a profound individual and societal impact. During the pandemic, governments implemented lockdowns to reduce the spread of the virus, forcing firms to close temporarily or individuals to work from home, imposing an economic burden on working-age individuals and causing productivity losses (1, 2). Due to generous job retention schemes, monthly unemployment rates declined to a lesser extent than number of hours worked in Europe (3, 4). Unemployment increased from 5.6% to 8.8% in OECD countries at the beginning of the pandemic (5), returning to around 5% throughout the pandemic (5). When jobs were not lost, hours worked decreased with an average reduction of 80 hours per worker in 2020 compared to 2019 in OECD countries, which recovered to a 22-hour reduction by 2022 (6). Sickness absence rates grew in the European Union from 9.4% in 2019-Q2 to 19% in 2020 (7). Regarding work quality, home workers with adequate conditions were able to enhance their work through increased flexibility and higher quality per hour worked (8, 9). However, increased demands and adaptation challenges adversely affected other workers (8, 10) for whom balancing work time with personal and family responsibilities decreased work quality.

The COVID-19 pandemic had a different impact on productivity indicators for subgroups, indicating the exacerbation of labor market inequalities of the pandemic (11, 12). Subsequent studies reported higher unemployment rates among women (13), those with children (14), in interpersonal occupations (14, 15), with (mental) health conditions (16), and among younger workers (12, 13, 17). Loss of hours was more prevalent among those with more precarious working conditions (12), lower income (12, 17), and workers with a lower education (17). Sickness absence was more prevalent among workers in healthcare and interpersonal occupations (18), and those with a lower education (18). Finally, loss of perceived work quality has been reported more frequently among those with worse mental health, lower education, and income (8).

Although the previous findings gain insight in the impact of the COVID-19 pandemic on productivity loss, most studies have investigated productivity loss by a single indicator. Moreover, most studies have been conducted cross-sectionally or considered relatively short periods of the pandemic. A comprehensive understanding of changes in productivity loss during the pandemic and its associated risk factors is important for effective comparison of productivity loss across groups and policy to reduce labor market inequality arisen during the pandemic. This requires longitudinal analysis, rather than observing specific periods like the beginning of the pandemic, or lockdown phases, and repeated measures of broad productivity loss indicators within the same sample to understand de differences in productivity loss.

Therefore, this study aimed to investigate productivity loss changes during the COVID-19 pandemic considering a comprehensive set of productivity loss indicators and to identify associated sociodemographic, health- and work-related factors.

## Methods

### Design and data source

In this study, we used data of the Work and Income module of the Lifelines COVID-19 cohort, which contained longitudinal measurements of indicators of work productivity, enriched with registry data from Statistics Netherlands (CBS) on income, and contract type. Lifelines is a multi-disciplinary prospective population-based cohort study examining in a unique three-generation design the health and health-related behaviors of 167 729 persons living in the north of The Netherlands. It employs a broad range of investigative procedures in assessing the biomedical, socio-demographic, behavioral, physical and psychological factors, which contribute to the health and disease of the general population, with a special focus on multi-morbidity and complex genetics. Additional information on the cohort and its add-ons is available elsewhere (19, 20). The Lifelines COVID-19 cohort consisted of add-on questionnaires conducted during the COVID-19 pandemic, including modules on work and income, COVID-19 infection, and mental health (21). The work and income module comprises information on changes in productivity indicators and occupational and financial status of participants. The data collection in Lifelines and its add-on studies was conducted according to the guidelines of the Declaration of Helsinki, and the Medical Ethics Committee of the University Medical Center Groningen approved all procedures (2007/152).

### Procedure

In March 2020, adult participants of the original cohort were invited to participate in the Lifelines COVID-19 cohort through online questionnaires. In total, 31 measurements took place between March 2020 and October 2022. Data was collected every week until June 2020, every two weeks until August of the same year, and every month until October 2022 (21). Waves 1 and 2 collectively form the baseline, containing basic socio-demographic, health, and work-related characteristics. Data on the work and income module was collected ten times (waves 8, 10, 13, 16, 19, 22, 23, 25, 27, and 29) starting in May 2020 and ending in October 2022. We linked these data on the individual level to registry data from CBS on income, and contract type, as has been done in other studies using Lifelines data (eg 23,).

### Inclusion criteria

**Figure 1:**
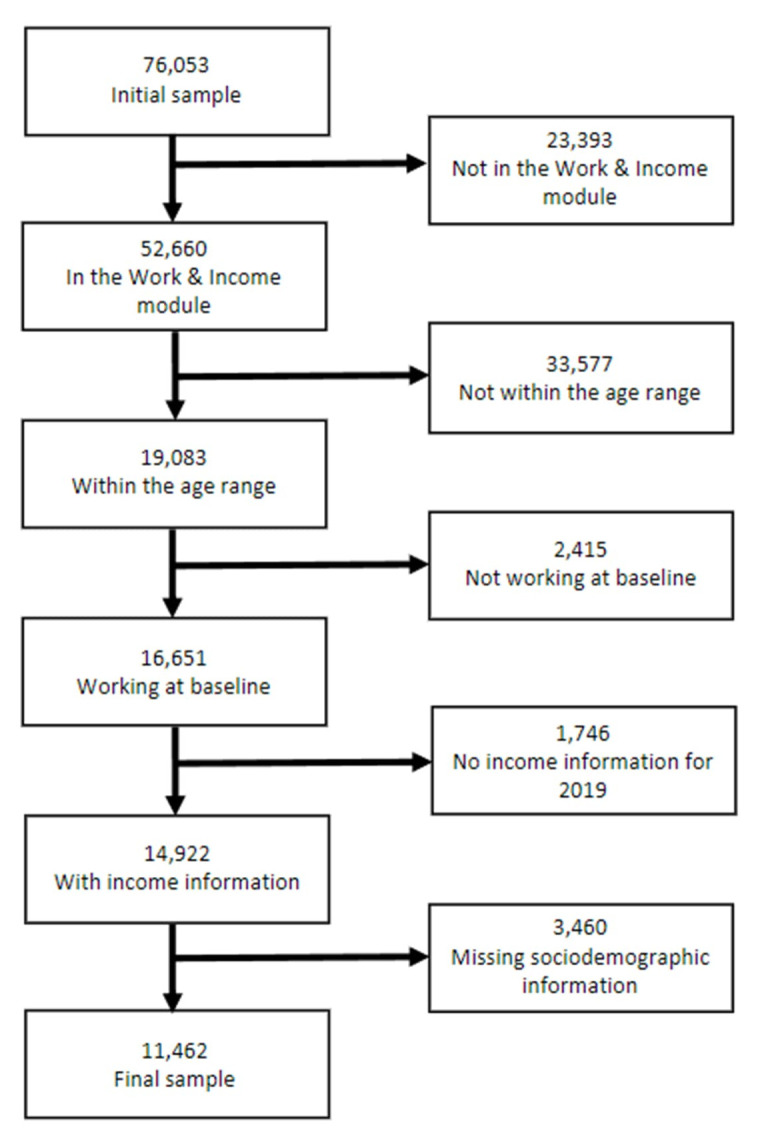
Flowchart of participant inclusion.

For this study, we included data of all working participants aged 18–64 years at baseline of the COVID-19 cohort who responded at least once to the work and income module. The reason to only include working participants at baseline is that we aimed to compare each participants’ productivity during the pandemic to their own pre-pandemic productivity. We could therefore not have a measurable reference point for the productivity of individuals who were not working at baseline. From the initial Lifelines COVID-19 cohort of 76 053 participants, exclusions were made for the following reasons: 23 393 were not part of the work and income module; 33 577 did not meet the age criteria at baseline; 2415 were not working at baseline; 1746 lacked income data from CBS; and 3460 were missing key sociodemographic or employment information (eg, household composition, critical job status). The final analytic sample included 11 462 participants. Figure 1 shows the participant inclusion flowchart.

### Productivity loss indicators

Productivity loss was measured as the reported change in four single productivity indicators compared to the beginning of the pandemic along four dimensions: unemployment, sickness absence, loss of work hours, and loss of quality of work. To evaluate changes in productivity during the pandemic, all indicators were compared to the period before the pandemic, albeit in different ways. Unemployment was assessed at each time point with the question “What do you currently do in your daily life?”, being considered unemployed when “unemployed” was answered. Sickness absence was measured with the question, “Have you called in sick or taken leave of absence in the last month?” and dichotomized into no/yes for each wave. Loss of hours was derived from the question, “How many hours per week did you work on average in the past month?”. This was combined with retrospective information asked at wave 8 (ie, “How many hours did you work in a regular week before the corona crisis?”). These questions were used to capture any reduction in hours at that point in time (no/yes). Finally, quality of work was assessed with the question “Is the quality of the work you are delivering worse, the same or better than before the corona crisis?”, measured on a 5-point Likert scale, with “worse” and “much worse” coded as a loss of quality during that time period. As all participants selected were working at baseline, going in unemployment or sickness absence marked a change during the study period. For loss of hours, currently worked hours at each time point were compared to hours worked before the pandemic. Finally, the quality of work question was relative to pre-pandemic work. As our outcome variables reflect productivity loss, they were coded to represent the presence or absence of a negative outcome (eg, reduction of hours versus no reduction of hours). This means they are only sensitive to negative changes in productivity, while neutral (eg, no change in hours) and positive changes (eg, increase in hours) are both coded the same (eg, no reduction of hours).

Ideally, productivity changes would be compared directly with pre-pandemic trends to capture the effects of the COVID-19 pandemic, ie, to establish whether these productivity losses were related to the COVID-19 pandemic or would have also occurred without the pandemic. However, comparable pre-pandemic data were not available in the regular Lifelines cohort for all outcomes in our cohort, particularly for sickness absence and perceived quality of work, which were only introduced in the Lifelines COVID-19 questionnaires. Additionally, pre-pandemic assessments of unemployment and hours worked in the regular Lifelines cohort were spaced at intervals too wide to allow for analysis of patterns or trends.

We therefore analyzed within-individual changes, comparing each participant’s outcomes over time to a pre-pandemic reference as directly asked in the Lifelines Covid-19 questionnaire. For several indicators, including sickness absence, hours worked, and quality of work, participants were asked to report on their pre-pandemic status. In the case of unemployment, our baseline measurement (March–May 2020) took place at a time when infection rates and government measures were still low in northern Netherlands and thus serves as a reasonable approximation for pre-pandemic employment status.

### Characteristics

Sociodemographic characteristics included age, educational level, household composition, and income level in 2019. Age was categorized into three work-life stages: 18–34 years (early); 35–49 years (mid); and 50–65 years (late). Educational level was classified as low, medium, or high. Household composition was assessed with the composite question “How many members of your household are between 0–12/13–18/19–35/35–50/51–65/60+ years of age?”. From this information, two binary variables were created denoting the presence or absence children (0–18 years), and adults (≥19 years) in the household or not having any household members, when the participants indicated zero members for all categories. Monthly household income was collected from CBS as the monthly gross income throughout 2019, from which the mean was calculated, and was categorized as low (≤€2000), medium (€2000–3000), and high (≥€3000).

Health-related characteristics include pre-existing health conditions, recent COVID-19, and ever having had COVID-19. Pre-existing health conditions was dichotomized into a no/yes variable based on the question “Do you have a chronic health condition?”. Participants were categorized as “yes” when they indicated the presence of any somatic or mental condition as in other papers using this dataset (eg, Ballering et al, 2022). Presence of COVID-19 was assessed by “Do you have, or have you had a coronavirus/COVID-19 infection?”, with only positive tests at official institutes being coded as yes. Participants who responded to a wave of data collection but did not indicate a COVID-19 infection were considered negative. At the end, two COVID-19 variables were created for each participant at each time point indicating recent COVID-19 and ever having had COVID-19 for an infection at any other previous point in time, to investigate a persistent effect. Those who indicated at least one instance of COVID-19 infection prior to each current wave were considered as positive for the latter variable.

Work-related characteristics included critical-job status, sector, and contract type. Critical job was measured with the question “Do you have a critical job (as defined by the government)?” (yes/no). This refers to occupations “that are essential to keep society running”. This includes, for example, healthcare providers, educators, public transport services, and those essential for the food chain. Sector was assessed using CBS data. From the 68 sector options, participants were grouped in 11 categories: industry, agri & food, construction, retail, hospitality, transport, education, health, other public, financial services, and other. Contract type was also assessed using data from Statistics Netherlands, treated as binary between permanent and non-permanent based on CBS guidelines.

### Statistical analysis

Baseline characteristics, as well as the prevalence of outcomes and time-varying variables at each wave are presented using descriptive statistics. Four generalized estimating equations (GEE) analyses, one for each outcome, were conducted to estimate the association of participants’ characteristics to productivity changes over time. GEE provides insight into within-person changes over time and between-person differences while accounting for the within-person response correlation.

We estimated the correlation structure of our GEE models in three steps. First, a simple correlation structure was plotted and interpreted manually. Second, the quasi information criterium (QIC) was estimated for our base model four times, one for each possible standard correlation structure (ie, exchangeable, independent, autoregressive, unstructured), omitting the missing values. Third, the QIC provides a simplified QIC (QICu) estimate on output, with the lowest value providing the most accurate fit for the model. All results pointed to the independent correlation structure, which was therefore used when estimating all our models. Each of the work productivity loss indicators was regressed separately against the sociodemographic, work-, and health-related characteristics within the GEE framework.

Additionally, a sensitivity analysis was conducted restricting all analyses to those who participated in wave 8. This was conducted due to the difference in sample for loss of hours compared to the other outcomes. Since loss of work hours requires a comparison to the retroactive question of hours worked before the pandemic, only asked at wave 8, the sample for this regression is limited to those present at wave 8, which could bias the estimates for the GEE if those missing in this wave differ from the rest of the population.

Data was handled and analyzed using the software package R, using haven, tidyverse, ggplot2, gee, geepack, and sjPlot.

## Results

### Descriptive statistics

Table 1 provides descriptives of the study sample, including the distribution of sociodemographic, health- and work-related characteristics and the attrition rate over time. Most (72.7%) of the sample were >50 years of age, and 65.5% were women. Regarding work-related characteristics, the majority (95.7%) had a permanent contract. Healthcare workers comprised 29.9% of the sample, 13.3% worked in education, and about half (51.7%) worked a critical job. Around a quarter (24.4%) reported some chronic health condition.

**Table 1:** Sociodemographic, health- and work-related characteristics (N=11 462).

Characteristics		Participants, N (%)
Gender		
	Male	3953 (34.5)
	Female	7509 (65.5)
Age (in work life stages)		
	Early life	455 (4.0)
	Mid life	2672 (23.3)
	Late life	8335 (72.7)
Education Level		
	Low	1350 (11.8)
	Middle	4736 (41.3)
	High	5376 (46.9)
Household members (children)		
	No	5281 (46.1)
	Yes	6181 (53.9)
Household members (adults)		
	No	1308 (11.4)
	Yes	10 154 (88.6)
Mean monthly income level (2019)		
	Low	3783 (33.0)
	Middle	3352 (29.2)
	High	4327(37.8)
Critical job		
	No	5537 (48.3)
	Yes	5925 (51.7)
Permanent contract		
	No	488 (4.3)
	Yes	10 974 (95.7)
Sector group		
	Industry	963 (8.4)
	Agri & food	270 (2.4)
	Construction	153 (1.3)
	Retail	952 (8.3)
	Transport	365 (3.2)
	Health	3421 (29.9)
	Education	1525 (13.3)
	Public other	1562 (13.6)
	Financial and services	1732 (15.1)
	Employment agencies	229 (2.0)
	Other	290 (2.5)
Chronic health condition		
	No	8669 (75.6)
	Yes	2793 (24.4)
Wave		
	8	8195 (71.5)
	10	6930 (60.5)
	13	7401 (64.6)
	16	6430 (56.1)
	19	6012 (52.5)
	22	4796 (41.8)
	23	4617 (40.3)
	25	4079 (35.6)
	27	3673 (32.0)
	29	4146 (36.2)

### Change in productivity loss indicators during the COVID-19 pandemic

Table 2 presents the findings on the change in productivity loss indicators across measurement waves. Unemployment remained low (<0.5%) throughout the study period. In contrast, the reduction of work hours peaked at 15% in March 2020, stabilizing just under 5% in October 2022. Sickness absence showed variability, changing with the course of the pandemic and spiking in September 2020 through March 2021, then again in February and September 2022. Similarly, loss of work quality varied and peaked at just under 5% before slowly declining back to 1%.

**Table 2:** Prevalence of time-varying characteristics and productivity indicators over time (N=11 462).

Wave	Unemployment N (%)	Sickness absence N (%)	Loss of work hours N (%)	Loss of quality N (%)	Recent COVID-19 N (%)	Previous COVID-19 N (%)
8	40 (0.3)	704 (6.1)	1719 (15.0)	481 (4.2)	25 (0.2	
10	33 (0.3)	409 (3.6)	943 (8.2)	300 (2.6)	<10	25 (0.2)
13	44 (0.4)	707 (6.2)	1285 (11.2)	284 (2.5)	<10	26 (0.2)
16	52 (0.5)	760 (6.6)	940 (8.2)	533 (4.7)	214 (1.9	28 (0.2)
19	43 (0.4)	683 (6.0)	966 (8.4)	508 (4.4)	435 (3.8	242 (2.1)
22	28 (0.2)	460 (4.0)	858 (7.5)	251 (2.2)	230 (2.0)	646 (5.6)
23	21 (0.2)	529 (4.6)	898 (7.8)	167 (1.5)	66 (0.6)	817 (7.1)
25	16 (0.1)	1000 (8.7)	856 (7.5)	193 (1.7)	1268 (11.1)	872 (7.6)
27	19 (0.2)	458 (4.0)	862 (7.5)	125 (1.1)	1441 (12.6)	2056 (17.9)
29	27 (0.2)	614 (5.4)	876 (7.6)	150 (1.3)	474 (4.1)	3142 (27.4)

### Associations between sociodemographic, health-, and work-related characteristics and changes in productivity loss indicators

Table 3 presents the GEE regression results. Women had higher odds of unemployment [odds ratio (OR) 1.32, 95% confidence interval (CI) 1.00–1.74] and sickness absence (OR 1.27, 95% CI 1.18–1.37) and lower odds of loss of work hours (OR 0.91, 95% CI 0.86–0.96). No differences in work quality were found compared to men. Those in the early working life stage had higher odds of requiring sickness absence (OR 1.47, 95% CI 1.28–1.69) and lose hours (OR 1.39, 95% CI 1.21–1.58) and quality of work (OR 1.98, 95% CI 1.65–2.37), despite having lower odds to become unemployed (OR 0.31, 95% CI 0.14–0.71), compared to older workers. Similar results were found for those in their mid-life stage, who had higher odds of requiring sickness absence (OR 1.41, 95% CI 1.32–1.51) and see decreased work hours (OR 1.12, 95% CI 1.06–1.19) and work quality (OR 1.43, 95% CI 1.31–1.56).

**Table 3:** Associations between productivity loss indicators and sociodemographic, work-, and health-related characteristics.[OR=odds ratio; CI=confidence interval; Obs=observations.]

		Unemployment N=11 459 Obs=56 148		Sickness absence N=11 334 Obs=54 060		Loss of work hours N=7312 Obs=41 079		Loss of quality N=10 876 Obs=47 029
		OR (95% CI)		OR (95% CI)		OR (95% CI)		OR (95% CI)
Gender								
	Male	Ref		Ref		Ref		Ref
	Female	1.32 (1.00–1.74)		1.27 (1.18–1.37)		0.91 (0.86–0.96)		1.04 (0.94–1.14)
Age (in work life stages)								
	Late life	Ref		Ref		Ref		Ref
	Mid life	1.00 (0.76–1.32)		1.41 (1.32–1.51)		1.12 (1.06–1.19)		1.43 (1.31–1.56)
	Early life	0.31 (0.14–0.71)		1.47 (1.28–1.69)		1.39 (1.21–1.58)		1.98 (1.65–2.37)
Education								
	High	Ref		Ref		Ref		Ref
	Mid	0.82 (0.63–1.06)		1.04 (0.98–1.11)		0.80 (0.76–0.85)		0.66 (0.60–0.72)
	Low	0.56 (0.38–0.84)		1.11 (1.01–1.23)		0.80 (0.73–0.87)		0.56 (0.47–0.66)
Average monthly income								
	High	Ref		Ref		Ref		Ref
	Mid	1.82 (1.28–2.59)		1.15 (1.07–1.24)		0.89 (0.83–0.94)		1.05 (0.95–1.15)
	Low	2.46 (1.72–3.52)		1.16 (1.07–1.26)		0.96 (0.90–1.03)		0.83 (0.74–0.93)
Household – Children								
	No	Ref		Ref		Ref		Ref
	Yes	0.68 (0.54–0.86)		1.04 (0.98–1.10)		0.85 (0.81–0.89)		1.09 (1.00–1.18)
Household – Adults								
	No	Ref		Ref		Ref		Ref
	Yes	1.01 (0.72–1.40)		0.89 (0.82–0.97)		1.10 (1.02–1.17)		1.05 (0.93–1.18)
Chronic health conditions								
	No	Ref		Ref		Ref		Ref
	Yes	1.01 (0.78–1.31)		1.45 (1.37–1.54)		1.03 (0.97–1.08)		1.30 (1.19–1.41)
Recent COVID-19								
	No	Ref		Ref		Ref		Ref
	Yes	1.23 (0.72–2.10)		3.59 (3.27–3.94)		1.26 (1.14–1.40)		1.47 (1.25–1.75)
Previous COVID-19								
	No	Ref		Ref		Ref		Ref
	Yes	0.60 (0.33–1.10)		1.05 (0.94–1.17)		1.08 (0.98–1.19)		1.24 (1.05–1.50)
Contract								
	Non-permanent	Ref		Ref		Ref		Ref
	Permanent	0.32 (0.22–0.47)		1.04 (0.89–1.22)		0.74 (0.65–0.84)		1.05 (0.83–1.33)
Sector group								
	Industry	Ref		Ref		Ref		Ref
	Agri and food	0.87 (0.41–1.84)		0.90 (0.72–1.11)		1.11 (0.93–1.33)		0.89 (0.58–1.37)
	Construction	0.24 (0.06–1.02)		0.80 (0.60–1.06)		0.76 (0.61–0.95)		0.36 (0.16–0.83)
	Retail	1.07 (0.69–1.66)		0.90 (0.78–1.04)		1.37 (1.21–1.54)		1.99 (1.54–2.57)
	Transport	0.74 (0.38–1.44)		0.81 (0.67–0.98)		1.84 (1.59–2.13)		1.69 (1.23–2.32)
	Health	0.31 (0.19–0.51)		1.01 (0.89–1.15)		1.20 (1.08–1.34)		2.27 (1.81–2.85)
	Education	0.37 (0.21–0.65)		1.04 (0.91–1.19)		1.54 (1.38–1.73)		2.63 (2.09–3.32)
	Public other	0.11 (0.05–0.26)		0.94 (0.82–1.06)		1.21 (1.09–1.34)		2.38 (1.90–2.98)
	Financial and services	0.78 (0.51–1.18)		0.82 (0.72–0.93)		0.98 (0.88–1.09)		1.67 (1.33–2.10)
	Employment agencies	1.54 (0.89–2.66)		1.12 (0.88–1.43)		1.15 (0.94–1.42)		2.11 (1.43–3.12)
	Other	0.92 (0.51–1.66)		0.82 (0.66–1.01)		1.96 (1.68–2.29)		2.41 (1.75–3.31)
Critical Job								
	No	Ref		Ref		Ref		Ref
	Yes	0.54 (0.40–0.71)		1.26 (1.18–1.34)		0.83 (0.79–0.88)		1.35 (1.23–1.48)

Those with mid- and low-education levels had lower odds to lose hours (OR 0.80, 95% CI 0.76–0.85; 0.80, 95% CI 0.73–0.87) and quality of work (OR 0.66, 95% CI 0.60–0.72; 0.56, 95% CI 0.47–0.66). Those with mid to low monthly income have higher odds of unemployment (OR 1.82, 95% CI 1.28–2.59; 2.46, 95% CI 1.72–3.52) and sickness absence (OR 1.15, 95% CI 1.07–1.24; 1.16, 95% CI 1.07–1.26). While those with mid-level income had lower odds of losing hours (OR 0.89, 95% CI 0.83–0.94), 95% CI those in the low-level had lower odds to reduce quality of work (OR 0.83, 95% CI 0.74–0.93).

Having children was negatively associated with being unemployed (OR 0.68, 95% CI 0.54–0.86) and losing hours of work (OR 0.85, 95% CI 0.81–0.89) and higher odds of loss of work quality (OR 1.09, 95% CI 1.00–1.18). Having a permanent contract was associated with lower odds of becoming unemployed (OR 0.32, 95% CI 0.22–0.47), as well as of reducing hours (OR 0.74, 95% CI 0.65–0.84). Those in a critical job had lower odds of unemployment (OR 0.54, 95% CI 0.40–0.71), and of losing hours of work (OR 0.83, 95% CI 0.79–0.88). However, they were at significantly higher odds of requiring sickness absence (OR 1.26, 95% CI 1.18–1.34) and reducing quality of work (OR 1.35, 95% CI 1.23–1.48). Participants with a chronic condition were at higher odds of sickness absence (OR 1.45, 95% CI 1.37–1.54) and of losing work quality (OR 1.30, 95% CI 1.19–1.41).

Those with a COVID-19 infection were at higher odds of requiring sickness absence (OR 3.59, 95% CI 3.27–3.94) and losing hours (OR 1.26, 95% CI 1.14–1.40) and quality of work (OR 1.47, 95% CI 1.25–1.75). In terms of working sector, those in health, education, and other public services had lower odds of becoming unemployed. Financial and services workers were at lower odds of claiming sickness absence. Higher odds of losing hours of work were found in most sectors compared to industry. The same was found for decrease in quality among workers in most sectors compared to industry.

### Sensitivity analysis

To examine the robustness of results when considering the participants who have missing information on work hours, we performed a sensitivity analysis looking only at the sample present in wave 8. When limiting the sample to those present in the first work and income measurement, only minor differences in direction, magnitude, and significance were encountered. Supplementary table S4 (www.sjweh.fi/article/4237) shows the results of the sensitivity analysis.

## Discussion

This study examined productivity loss changes during the COVID-19 pandemic by considering a comprehensive set of productivity loss indicators measured ten times from May 2020 to October 2022 in a population-based cohort in the north of the Netherlands. The findings point to productivity loss at all time points during the pandemic for hours worked, sickness absence and work quality.

In our study population, unemployment remained low during the pandemic, with <1% being out of work at any given point. This could be a consequence of the generous work support measures in The Netherlands, which offered support to companies and secured many jobs despite the crisis (3, 22). In contrast, the productivity indicators hours worked, quality of work, and sickness absence showed more substantial adverse changes, with magnitudes fluctuating during the pandemic. These indicators peaked during lockdown periods and increased infection rates, such as in March and December 2020. This is in line with the current literature, pointing to pandemic-related factors increasing fatigue among workers, leading to decreased work quantity and quality (8, 23), and COVID-19 itself causing sickness absence (18). This represents a subtle but important consequence of COVID-19, that is, despite the success of government measures to retain employment, there has been productivity loss across different aspects of quantity and quality of work, with upwards of 15% of our sample having reduced work hours.

Regarding the association of changes in productivity indicators with sociodemographic, health- and work-related characteristics, we identified two distinct sets of characteristics with similar patterns. One set of characteristics pertains to socioeconomic indicators (income, education). Those with low education and income have been established in the literature as being at risk for labor market shocks (24), especially during times of crisis. Our results point partially similar direction, as those with lower income were at higher odds of becoming unemployed. These results are not shared by those with lower education, which had lower odds of becoming unemployed. Both those with lower and mid-level income were more likely to require sickness absence, which could be related to the higher concentration of occupations that require contact in this income range, leading to higher exposure to COVID-19 infection risk, as suggested in the literature (25). In addition, we see those with mid- to lower-level education at lower odds for losing hours and quality.

A second set of characteristics pertains to contextual factors (family composition, critical jobs). When looking at family composition, we see a lowered odds of becoming unemployed and losing hours among those with children. Despite going against the expectations of current literature, this might be a compensatory effect, as these participants might be more pressured to maintain their jobs and hours. Studies have shown worse impacts of job insecurity during the pandemic on working parents (26), which could lead to increased commitment and effort to maintain employment. While the government support measures could act as a safety net for parents, the perceived medium and long-term uncertainty about their duration or continuity throughout the pandemic, combined with the financial and caregiving responsibilities associated with having dependents, could partly explain why parents were less likely to experience reductions in employment or hours worked during this period. Looking at health characteristics, we see recent COVID-19 and chronic conditions being associated with increased odds of sickness absence and work quality loss. The literature has already pointed to the impacts of (mental) health conditions on work productivity even before the pandemic (27, 28), which could have been exacerbated by the increased barriers to access healthcare during the pandemic (29), leading to increased sickness absence and reduction in work capacity. Those in critical occupations were, as expected, less likely to lose jobs and hours, but were more likely to require sickness absence and lose work quality. This is in line with the literature as the interpersonal nature of these occupations requires exposure to health risks (18).

### Strengths and limitations

This study had several strengths. First, it had a longitudinal design with repeated measurements for each indicator of productivity with over two and a half years follow-up. This permitted this study to follow not only the prevalence of productivity loss indicators at any point, but their change over time. Second, multiple indicators of productivity were used, providing information on different indicators in the same sample to provide a comprehensive picture of productivity changes. Third, it utilized a large, population-based cohort from the north of the Netherlands, which, in addition to providing a large sample, also allows us to look at a general working population. Finally, despite the risk of selection bias in one of the analysis due to reduced sample size, the results proved robust in the sensitivity analysis.

This study also had some limitations. First, our sample had an older average age in comparison to the general Lifelines population, which in turn is older than the general Dutch population (19). We also find an overrepresentation of permanent contracts as well as of some sectors, such as healthcare compared to the general Lifelines population. As the literature in The Netherlands point to a more significant employment loss for those with flexible or temporary contracts during the pandemic (30), the impacts on productivity loss might be underestimated in this study. Second, most of the characteristics used to estimate associations to the productivity indicators were fixed at baseline, which means that this study did not account for some life events during the study period, such as switching of jobs or changing chronic health status. This could mask some effects of the pandemic, as workers who were initially in more affected areas might have switched career tracks, lessening their productivity loss in later waves. In addition, information on the productivity indicators was composed entirely of self-reported measurements. Combining self-reported and registry-based data on productivity might have strengthened our analyses by improving robustness. While some indicators, ie, hours worked and employment status, could be found in registry data, other key indicators, such as quality of work and absenteeism, are subjective or not available in registry data. Thus, to ensure a consistent and comprehensive measurement of productivity across all participants, we chose to derive all productivity indicators from the same source, the Lifelines COVID-19 questionnaires. Although self-reports may potentially introduce information bias (31), a recent study on earnings and work hour dynamics for The Netherlands concluded that observed pattern were similar for registry data and self-reported data (32). Finally, although the Lifelines COVID-19 cohort is part of a large-scale generally representative populational cohort, it is not representative of the Dutch population. As is reported in previous research (21), responders of the Lifelines COVID-19 cohort are older and more often female. Having an older population may have influenced our results concerning the prevalence of sickness absence being overreported, as older workers in The Netherlands have on average more frequent and longer episodes of sickness absence than younger workers (33). Similarly, the overrepresentation of women may reflect a higher proportion of part-time employment and a concentration in specific sectors such as health care and education, which were affected differently by pandemic-related restrictions. We also observed an overrepresentation of individuals with permanent contracts and certain sectors. Since existing literature in The Netherlands points to a more significant employment loss for those with flexible or temporary contracts during the pandemic (30), our estimates of productivity loss might be underestimated in this study if not controlled for type of contract. However, sociodemographic, work-, and health-characteristics were controlled for in our main analyses. Therefore, the results from our regression models are robust to imbalances in the sample.

### Concluding remarks

In conclusion, despite low unemployment rates during the COVID-19 period, productivity loss was observed when looking at other productivity indicators, ie, sickness absence, loss of hours and quality of work. Productivity was also not lost in an equal manner among different socioeconomic groups. Our findings suggests that those with lower socioeconomic were at higher risk of becoming unemployed, potentially pointing to limitations in the reach of government support schemes. Meanwhile, critical workers and younger workers, kept their jobs, but were more likely to become sick and lose quality of work. When preparing for future crises, attention should be paid to multiple aspects of productivity loss and their associated risk factors through targeted interventions to higher risk groups to protect workers and minimize losses in productivity.

## Supplementary material

Supplemenary material
