# Supplementary material for: Productivity changes during the COVID-19 pandemic and its associated risk factors
Source: Scand J Work Environ Health. 2025 Aug 29;51(5):394–403. doi: 10.5271/sjweh.4237 (PMC12414509; doi:10.5271/sjweh.4237)
Supplement: Supplemenary material [file SJWEH-51-394-S001.pdf]

Productivity changes during the COVID-19 pandemic and its associated risk factors[1]

by Guilherme Monteiro Sanchez Dalla Riva, MSc,[2] *Sander KR van Zon, PhD, Patricia Ots, PhD, Gerard van den Berg, PhD, Lifelines Corona Research Initiative, Sandra Brouwer, PhD, Raun van Ooijen, PhD*

1. Supplementary material
2. Correspondence to: Guilherme MS Dalla Riva, Department of Health Sciences, Community and Occupational Medicine, University of Groningen, University Medical Center Groningen, Antonius Deusinglaan 1, 9700 RB Groningen, The Netherlands. [E-mail: g.m.sanchez@umcg.nl]

Table S1. Associations between productivity loss indicators and sociodemographic, work-, and health-related characteristics using only those that responded to Wave 08

	**Unemployment OR (95% CI)**	**Sickness Absence OR (95% CI)**	**Loss of Work Hours OR (95% CI)**	**Loss of Quality OR (95% CI)**
**Gender**				
Male	*Ref*	*Ref*	*Ref*	*Ref*
Female	1.46 (1.06 – 1.99)	1.28 (1.18 – 1.38)	0.91 (0.86 – 0.96)	1.07 (0.96 – 1.19)
**Age (in work life stages)**				
Late Life	*Ref*	*Ref*	*Ref*	*Ref*
Mid Life	0.92 (0.67 – 1.284)	1.44 (1.34 – 1.55)	1.12 (1.06 – 1.19)	1.36 (1.24 – 1.50)
Early Life	0.23 (0.09 – 0.63)	1.52 (1.30 – 1.78)	1.39 (1.21 – 1.58)	1.84 (1.49 – 2.26)
**Education**				
High	*Ref*	*Ref*	*Ref*	*Ref*
Mid	0.86 (0.64 – 1.15)	1.06 (0.98 – 1.14)	0.80 (0.76 – 0.85)	0.66 (0.60 – 0.73)
Low	0.59 (0.38 – 0.91)	1.14 (1.02 – 1.27)	0.80 (0.73 – 0.87)	0.62 (0.52 – 0.75)
**Average Monthly Income**				
High	*Ref*	*Ref*	*Ref*	*Ref*
Mid	1.75 (1.18 – 2.61)	1.18 (1.09 – 1.28)	0.89 (0.83 – 0.94)	1.06 (0.95 – 1.18)
Low	2.50 (1.67 – 3.73)	1.17 (1.07 – 1.28)	0.96 (0.90 – 1.03)	0.83 (0.73 – 0.94)
**Household – Children**				
No	*Ref*	*Ref*	*Ref*	*Ref*
Yes	0.56 (0.43 – 0.73)	1.05 (0.98 – 1.12)	0.85 (0.81 – 0.89)	1.06 (0.97 – 1.16)
**Household – Adults**				
No	*Ref*	*Ref*	*Ref*	*Ref*
Yes	1.22 (0.84 – 1.78)	0.86 (0.79 – 0.94)	1.10 (1.02 – 1.17)	1.08 (0.95 – 1.23)
**Chronic Health Conditions**				
No	*Ref*	*Ref*	*Ref*	*Ref*
Yes	0.99 (0.74 – 1.32)	1.44 (1.35 – 1.54)	1.03 (0.97 – 1.08)	1.29 (1.17 – 1.42)
**Recent COVID-19**				
No	*Ref*	*Ref*	*Ref*	*Ref*
Yes	1.36 (0.76 – 2.46)	3.74 (3.36 – 4.15)	1.26 (1.14 – 1.40)	1.48 (1.22 – 1.80)
**Previous COVID-19**				
No	*Ref*	*Ref*	*Ref*	*Ref*
Yes	0.76 (0.40 – 1.44)	1.08 (0.96 – 1.22)	1.08 (0.98 – 1.19)	1.27 (1.04 – 1.55)
**Contract**				
Non-Permanent	*Ref*	*Ref*	*Ref*	*Ref*

Permanent	0.29 (0.19 – 0.43)	1.06 (0.89 – 1.26)	0.74 (0.65 – 0.84)	1.10 (0.85 – 1.44)
**Sector Group**				
Industry	*Ref*	*Ref*	*Ref*	*Ref*
Agri and Food	0.99 (0.44 – 2.20)	0.89 (0.70 – 1.14)	1.11 (0.93 – 1.33)	1.04 (0.65 – 1.68)
Construction	0.30 (0.07 – 1.28)	0.79 (0.58 – 1.08)	0.76 (0.61 – 0.95)	0.48 (0.21 – 1.11)
Retail	1.06 (0.64 – 1.74)	0.88 (0.78 – 1.04)	1.37 (1.21 – 1.54)	1.95 (1.44 – 2.63)
Transport	0.51 (0.22 – 1.18)	0.80 (0.75 – 0.99)	1.84 (1.59 – 2.13)	1.71 (1.18 – 2.48)
Health	0.27 (0.16 – 0.48)	1.03 (0.90 – 1.19)	1.20 (1.08 – 1.34)	2.59 (1.99 – 3.38)
Education	0.32 (0.17 – 0.62)	1.01 (0.87 – 1.18)	1.54 (1.38 – 1.73)	2.98 (2.27 – 3.91)
Public Other	0.12 (0.05 – 0.30)	0.97 (0.84 – 1.13)	1.21 (1.09 – 1.34)	2.63 (2.02 – 3.41)
Financial and Services	0.86 (0.54 – 1.38)	0.81 (0.70 – 0.93)	0.98 (0.88 – 1.09)	1.76 (1.34 – 2.30)
Employment Agencies	1.56 (0.85 – 2.85)	1.16 (0.88 – 1.51)	1.15 (0.94 – 1.42)	2.35 (1.50 – 3.68)
Other	0.76 (0.39 – 1.51)	0.82 (0.65 – 1.04)	1.96 (1.68 – 2.29)	2.70 (1.89 – 3.87)
**Critical Job**				
No	*Ref*	*Ref*	*Ref*	*Ref*
Yes	0.60 (0.44 – 0.82)	1.28 (1.19 – 1.38)	0.83 (0.79 – 0.88)	1.37 (1.23 – 1.52)
**Participants**				
N	8,193	8,117	7,312	7,916
Observations	47,719	45,906	41,079	39,926
